# Functional food: complementary to fight against COVID-19

**DOI:** 10.1186/s43088-022-00217-z

**Published:** 2022-03-07

**Authors:** Maisha Farzana, Sagarika Shahriar, Faria Rahman Jeba, Tahani Tabassum, Yusha Araf, Md. Asad Ullah, Jarin Tasnim, Agnila Chakraborty, Taslima Anjum Naima, Kay Kay Shain Marma, Tanjim Ishraq Rahaman, Mohammad Jakir Hosen

**Affiliations:** 1 Biotechnology Program, Department of Mathematics and Natural Sciences, School of Data and Sciences, Brac University, Dhaka, Bangladesh; 2 Department of Genetic Engineering and Biotechnology, School of Life Sciences, Shahjalal University of Science and Technology, Sylhet, Bangladesh; 3 Department of Biotechnology and Genetic Engineering, Faculty of Biological Sciences, Jahangirnagar University, Savar, Dhaka, Bangladesh; 4 Department of Pharmacy, Faculty of Biological Sciences, University of Chittagong, Chattogram, Bangladesh; 5 Department of Biotechnology and Genetic Engineering, Faculty of Life Sciences, Bangabandhu Sheikh Mujibur Rahman Science and Technology University, Gopalganj, Bangladesh

**Keywords:** Functional food, Immune system, Vitamins, Medicinal plants, Probiotics

## Abstract

**Background:**

The novel coronavirus has embarked on a global pandemic and severe mortality with limited access for its treatments and medications. For the lack of time, research, and enough efficacy, most vaccines are underdeveloped or unreachable to society. However, many recent studies suggest various alternative, complementary remedies for COVID-19, which are functional foods. This review provides an overview of how functional foods can play a great role through modulating the host immune system, generating antiviral activities, and synthesizing biologically active agents effective against the coronavirus.

**Main body:**

This review article summarizes the natural defense mechanisms in tackling SARS-CoV-2 alongside conventional therapeutic options and their corresponding harmful side effects. By analyzing bioactive components of functional foods, we have outlined its different contributions to human health and its potential immunomodulatory and antiviral properties that can enhance resistivity to viral infection. Moreover, we have provided a myriad of accessible and cost-effective functional foods that could be further investigated to target specific key symptoms of COVID-19 infections. Finally, we have found various functional foods with potent bioactive compounds that can inhibit or prevent COVID-19 infections and disease progression.

**Short conclusion:**

Numerous functional foods can help the body fight COVID-19 through several mechanisms such as the reduced release of pro-inflammatory cytokines, reduced expression of ACE2 receptors in cells, and inhibiting essential enzymes in SARS-CoV-2.

## Background

The pandemic “Coronavirus Disease 2019” (COVID-19) is caused by Severe Acute Respiratory Syndrome Coronavirus 2 (SARS-CoV-2) and was officially identified in late December 2019 at Wuhan, China. COVID-19 is highly contagious and mainly characterized by mild to severe respiratory infections in humans [1]. SARS-CoV-2 is mainly spread by respiratory droplets of an infected individual while coughing, sneezing, and talking. As of 10th February 2022, more than 404 million cases with over 5.5 million deaths have been reported worldwide [2]. Moreover, many countries of the world have faced several waves of reoccurring pandemic with different mutated strain of this virus [3].

The vaccine is the ultimate target to prevent and control COVID-19. Currently, there are 13 authorized vaccines available against SARS-CoV-2 with an accuracy of 75–95% [4, 5]. However, as of February 2022, a total of 10.1 billon vaccine doses have been administered [6]. Every country of the world is looking for these vaccines, but due to urgent demand compared to production capacity, high price, less efficacy, and possible side effects, it may take years to ensure vaccination [7, 8]. As a result, most affected countries implemented complimentary restrictive measures, including wearing masks, social distancing, and lockdowns to slow down the spread of this virus.

Moreover, synthetic antiviral drugs are also underdeveloped, expensive, might have adverse impacts, and are only used in selected situations to reduce the duration of illness [9–11]. Thus, it necessitates alternative, complementary remedies for COVID-19. The rapid changes in the strains of SARS-CoV-2 have led to diversified forms of medications and dietary profiles for controlling and preventing the infection risk [12]. Functional foods can play a great role through modulating the host immune system, generating antiviral activities within the host, and synthesizing biologically active agents effective against the current COVID-19 pandemic [13]. This review featured the possible pathomechanism of SARS-COV-2 and the mechanisms of functional food to fight against it.

## Pathomechanism of COVID-19

The pathogenesis of COVID-19 is still not fully understood. SARS-CoV-2 is primarily transmitted by micro-droplets exhaled by infected individuals or fomites and bind with the ACE2 receptor to get entry to lung cells, and then the virus targets type-II ACE2^+^ alveolar cells present in the lungs [14]. The infected lung cells secrete cytokine IL-8, which attracts T lymphocytes and neutrophils [15]. The innate immune system initially attempts to defend the body in various ways: mucosa-associated lymphoid tissues (MALT) protect mucosal surfaces, and Pattern Recognition Receptors (PRR) such as TLR7 recognize pathogen-associated molecular patterns (PAMPS). Additionally, activation of transcription factors such as nuclear factor κB (NF-κB) and activator protein 1 (AP-1) results in the release of pro-inflammatory cytokines such as IL-6, TNF-α, and interferon 1 [16–18]. Due to the secretion of various chemokines, innate immune cells accumulate in the area of infection, which releases even more chemokines and recruit lymphocytes leading to antigen presentation via dendritic cells [19–21].

**Fig. 1:**
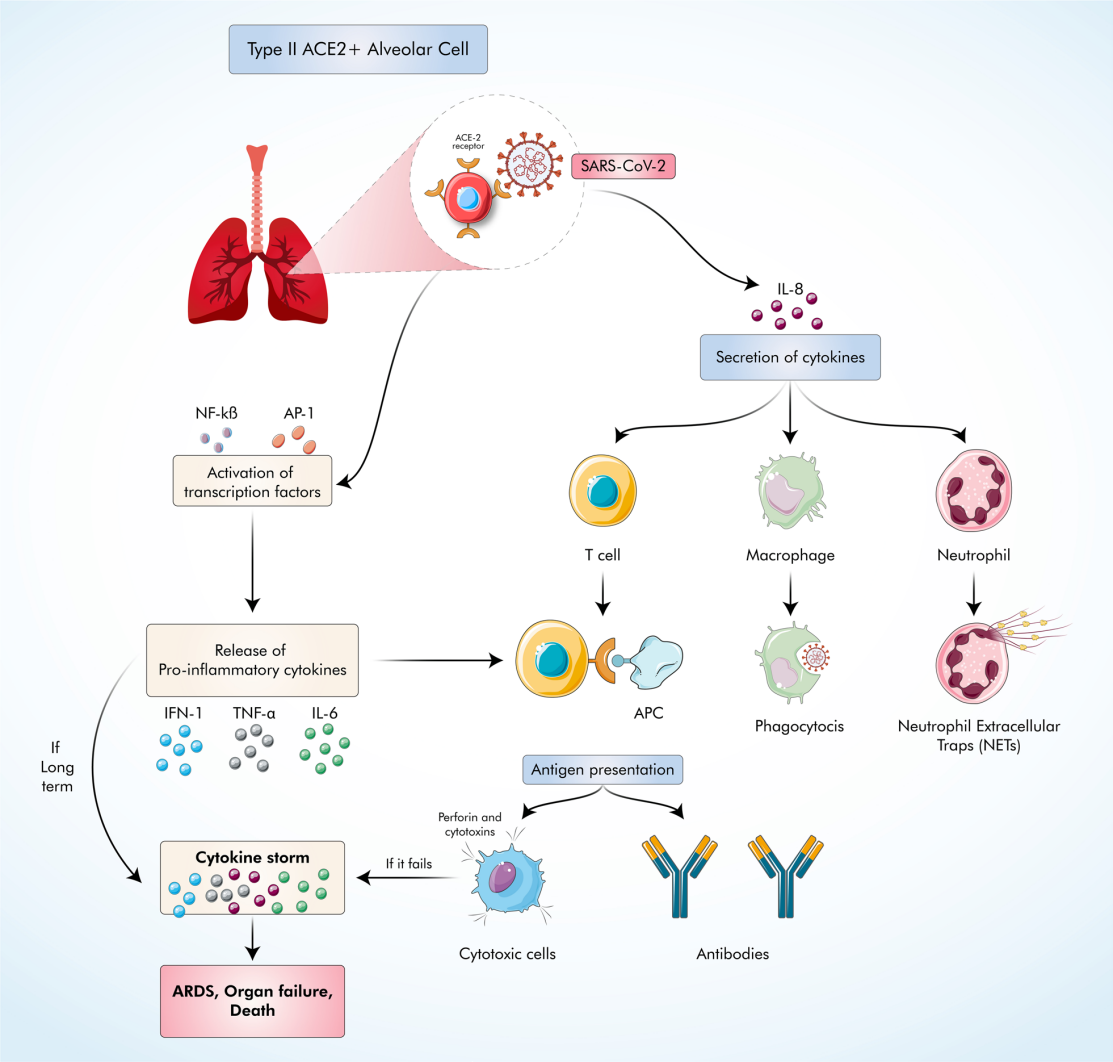
SARS-CoV-2 infects primarily alveolar cells through the ACE2 receptor. The immune system responds to the virus by activating different transcription factors that release pro-inflammatory cytokines that trigger the accumulation of immune cells leading to antigen presentation. Additionally, secretion of specific cytokines such as IL-8 attracts neutrophils and macrophages to the site of infection alongside T lymphocytes. While neutrophils kill by oxidative burst and NETs with macrophages inducing phagocytosis, CD4 + T cells help to activate B cells producing anti-SARS-CoV-2 and CD8 + T cells kill infected cells through the release of perforins and cytotoxins. If CD8 + T cells fail to kill the virus in the presence of inflammatory cytokines over the long term, cytokine storm occurs, leading to disease progression and complications such as ARDS, organ failure, and death

Meanwhile, neutrophils at the infection site attempt to eliminate the virus by using neutrophil extracellular traps (NETs), phagocytosis, and oxidative burst [22]. Antigen presentation starts the phase of adaptive immune response where it depends on T lymphocytes. Helper T cells stimulate B cells to produce SARS-Cov-2-specific antibodies, while Cytotoxic T cells remove infected cells. Research has shown that 80% of cell infiltration is done by Cytotoxic T cells in COVID-19 patients [23]. If Cytotoxic T cells fail to eliminate infected cells and the disease progress for a longer time, the release of pro-inflammatory cytokines becomes uncontrollable, leading to a cytokine storm. Ultimately, multiple complications such as acute respiratory distress syndrome (ARDS) and organ failure [24] occur in infected patients, which can eventually progress to death (Fig. 1). The overall process is summarized in Fig. 1.

## Functional food and its essential components and properties

Functional foods, also known as nutraceuticals, are defined as foods containing bioactive compounds that have beneficial effects on consumer health. Bioactive compounds are phytochemicals extracted and consumed as supplements or may have medicinal value when engulfed as whole food. Functional food has gained popularity to prevent numerous diseases, boost growth, and enhance host metabolic activity [25, 26]. In addition, such functional foods may be useful for preventing non-communicable diseases like COVID-19.

### Essential components of functional foods

#### Multivitamins

Functional food has several essential components, including vitamins. Vitamins are organic compounds required in the diet for the growth and development of the body. Serving countless functions in the body, deficiency of vitamins can hinder the proper functioning of the body and contribute to improper immune responses to infections. A summary of essential vitamins and their rules in the body found in different functional foods is summarized in Table 1.

**Table 1:** Different vitamins found in different functional foods and their beneficial properties outlined

Type	Daily recommended intake in adult individuals (19–50 years)	Sources of functional food	Features	Functions	References
Vitamin A	900 μg/day for men, 700 μg/day for women	Eggs, fish oil, fish liver, milk, corn, yam, sweet potato, carrot, lima bean, sorghum, green soybeans	Antioxidant, fat-soluble	Helps in antibody response (particularly IgA); develops type 1 and type 2 helper T cells; prevents cancer by protecting DNA from damage by free radicals; maintains epithelium of the cornea; acts as a precursor to visual pigments	[27–29]
Vitamin B6	1.3 mg/day for both men and women	Potato, white rice, chicken, beef, grains, legume, fish, non-citrus fruits, banana, onion, pineapple	The enzymatic cofactor, water-soluble, highly reactive during phosphorylation, confer some antioxidant properties	Involved in the maturation of lymphocytes; required for the synthesis of antibodies and cytokines; enhances immune response; could potentially reduce hypertension; required for the metabolism of amino acids, sugars, and fatty acids	[27, 30–34]
Vitamin B12	2.4 μg/day for both men and women	Beef, whole milk, egg, shellfish, turkey, mutton, soybean, tea drink, fish sauce, fermented fish, cheese	The enzymatic cofactor, water-soluble, may potentially confer antioxidant properties	Required for synthesis of antibodies and cytokines; involved in erythrocyte maturation; benefits cardiovascular system through the reduction in homocysteine; needed for healthy bones; increases the abundance of cytotoxic T cells	[27, 32, 33, 35, 36]
Folate	300–400 μg/day for both men and women	pasta, bean, green salad, orange juice, peanut, spinach, lentil, parsley, beets, romaine lettuce, Brussels sprouts	The enzymatic cofactor, water-soluble, reduced form has similar antioxidant property as Vitamin C and E	Regulates immune response by type 1 helper T cells; improves the cytotoxic function of NK cells; needed by regulatory T cells in the small intestine; methylates cytosine in DNA; produces purines and pyrimidines	[27, 32, 33, 35, 37–39]
Vitamin C	90 mg/day for men, 75 mg/day for women	Guava, oranges, lemon, broccoli, papaya, red bell pepper, pineapple, blackberry, black currant, strawberry, tomato	Enzymatic cofactor, water-soluble, antioxidant	Boosts collagen production; promotes differentiation of B and T lymphocytes; eliminates pathogens and assists in the production of antibodies; involved in vasodilation; protects DNA from oxidative damage	[27, 40–43]
Vitamin D	15 μg/day for both men and women	Egg yolk, tuna, mushroom, soy milk, cow milk, sardines, orange juice, salmon, eel, cod, butter	Antioxidant, Fat-soluble	Helps to stimulate an innate immune response; enhances antigen processing; supports differentiation of monocytes to macrophages; maintains calcium concentration in extracellular fluid; required for strong bones	[27, 33, 44]
Vitamin E	15 mg/day for both men and women	Sunflower seeds, asparagus, shrimp, avocado, olive oil, almond, raspberry, spinach, broccoli, bell peppers, carrot	Antioxidant, Fat-soluble	Increases IL-2 levels; protects the body from oxidative damage; metabolizes arachidonic acid; stabilizes lipid bilayer of cell membranes; regulates protein kinase C	[27, 33, 45, 46]

#### Minerals

Minerals are inorganic, required in small amounts in the body for various functions, including adequate functioning of the immune system [47]. Some minerals are required in larger amounts, e.g., calcium, phosphorus, magnesium, sodium, potassium, and chloride. Others are required in minute amounts, also known as trace minerals, including zinc (Zn), copper (Cu), selenium (Se), and iron (Fe), serve critical roles in many biochemical processes (Table 2). Similar to vitamins, a deficiency in such elements hampers health [47]. Minerals, both abundant and trace, and their contributions to the immune system are outlined in Table 2.

**Table 2:** Different minerals and their beneficial properties outlined

Mineral	Daily recommended intake in adult individuals (19–50 years)	Sources	Functions	References
Zinc	11 mg/day for men, 8 mg/day for women	Viscera, legumes, nuts, red meat, milk, eggs, cheese, cereals	Serves as a cofactor for metalloenzymes that repair cell membranes in innate barriers; helps in antibody production; enhances NK cell cytotoxic activity and phagocytic activity of macrophages and monocytes; helps in differentiation of immune cells; improves resistance to infections; helps in wound healing	[27, 47, 48]
Iron	8 mg/day for men, 18 mg/day for women	Viscera, legumes, nuts, red meat, eggs, seafood, potatoes	Regulates ratio between helper T and cytotoxic T cells; helps in IFN-γ production; essential for enzymes in immune cells; improves the growth of epithelial tissue in innate barriers	[27, 48]
Copper	900 μg/day for both men and women	Viscera, legumes, cheese, seafood, nuts, poultry, rabbit	Fights infectious agents in phagolysosomes of macrophages; needed to produce IL-2 and differentiation of T cells; improves NK cell activity; needed for monocytes and neutrophils to function	[27, 48]
Selenium	55 μg/day for both men and women	Sea salt, eggs, bread, mushroom, garlic, asparagus, nuts	Improves IFN-γ production; increases Th cells; maintains antibody levels; helps in differentiation of T lymphocytes; improves immune response to viruses; essential for the function of NK cells and leukocytes	[27, 49]
Magnesium	400–420 mg/day for men, 310–320 mg/day for women	Almonds, bananas, black beans, broccoli, brown rice, cashews, flaxseed	Helps in antigen binding to macrophages; modulates leukocyte activation; protects DNA from oxidative damage; cofactor in antibody production; required in antibody-dependent cytolysis	[27, 50]

#### Nutraceuticals supplements

Nutraceuticals, derived from “nutrition” and “pharmaceutics,” are substances used to improve health, slow down aging, enhance life expectancy, protect from chronic illnesses or help the body functions [51]. Nutraceutical products originate from herbs, diet supplements, particular diets, and processed foods. Research has indicated the successful application of nutraceutical products for treating various disorders such as diabetes, atherosclerosis, CVDs, cancer, and neurological disorders (Table 3) [51]. While a myriad of nutraceutical supplements is available on the market, specific nutraceutical supplements are provided in Table 3 to summarize the overall benefits of nutraceuticals.

**Table 3:** Selected nutraceutical supplements with their associated benefits summarized

Nutraceutical supplement	Source (s) rich in the nutraceutical compound	Functions	References
Quercetin	Onion, red grapes, broccoli, citrus fruits	Protects blood vessels from oxidative stress and low-density lipoprotein	[51, 52]
Omega-3 fatty acids	Fish	Treats eye disorders such as age-related macular degeneration (AMD); prevents heart damage in diabetic patients	[51]
Cod liver oil	Cod liver	Reduces symptoms of cardiovascular-related conditions	[51, 53]
Ginseng	Panax ginseng, Panax quinquefolius	Protects against infections; delays osteoporosis; prevents and treats autoimmune diseases	[54]
Lutein	Kale, spinach, corn, eggs	Combats oxidative stress; delays Alzheimer's disease; treats visual disorders such as AMD	[51, 52]

#### Probiotic supplements

Probiotics are live microorganisms that provide benefits to the host when ingested in adequate amounts [55]. Strains belonging to *Lactobacillus* and *Bifidobacterium* sp. are commonly used as probiotic preparations in fermented milk and yogurt. Probiotics maintain a healthy balance between pathogenic and non-pathogenic bacteria alongside pro-inflammatory and anti-inflammatory cytokines [51]. Furthermore, probiotics improving gut microbiome affect viral vaccine responses [56]. Therefore, probiotics might play a significant role in fighting COVID-19 by mediating the immune response of the infected patients.

### Properties of functional food

#### Immunomodulatory Properties

Functional foods play immunomodulatory roles by improving both adaptive and innate immune responses, which are imperative to the viability of living organisms [26]. Additionally, functional foods facilitate the release of immunostimulatory chemicals like cytokines and stimulate TNF-α and macrophage release that strengthen the body’s defense [57, 58]. Various explanations have been proposed for these effects, including the interaction of immunomodulatory peptides and opioid receptors on the immune cell surface, presence of arginine at C-terminal of peptides similar to ACE-inhibitory peptides, and activation of reactive oxygen species [58]. Consequently, deficiencies in diet had negative consequences on immune system functions suggesting immunomodulatory functions of these nutrients. For example, protein-energy malnutrition (PEM) had reduced lymphocyte proliferation and opsonic plasma activity [59]. In other instances, reduced zinc levels resulted in lower cytokine concentration in plasma alongside less proliferation of T cells, while a vitamin B6 deficiency led to a decrease in antibody-forming cells [60, 61].

#### Antiviral activities

The antiviral properties of functional foods have also been documented to be effective against different viruses. Glycyrrhizin is effective against Japanese encephalitis virus, Human immunodeficiency virus type 1 (HIV-1), and chronic hepatitis C virus (HCV) [62]. Glycyrrhizin in licorice roots has been shown to inhibit SARS-CoV replication as it affects cellular signaling pathways and induces nitrous oxide synthesis in macrophages, preventing the virus from replicating [12]. Epigallocatechin gallate (EGCG), found in green tea and its ester derivatives, has proven to inhibit the NS3/4A protease enzyme responsible for activating functional proteins in HCV [63]. Moreover, EGCG derivatives inhibit an α-glucosidase enzyme that prevents glycoprotein synthesis for the viral envelope [63]. Sulfated polysaccharides in seaweed compete with viruses for the binding site on cells and prevent their entry by synergistic interaction with the cell [64]. Therefore, consumption of seaweed is shown to be effective against Herpes simplex virus Types 1 and 2 (HSV-1 and HSV-2), human cytomegalovirus (HCMV), HIV-1, respiratory syncytial virus (RSV), influenza virus, and bovine viral diarrhea virus [64].

#### Additional features

In addition to immunomodulatory and antiviral properties, functional foods also have antibacterial, antifungal, antioxidant, anticancer, anti-inflammatory, and neuroprotective properties [65–75].

## Currently available non-specific treatment options for COVID-19 and their side effects

Several domestic and foreign companies are using various technologies to develop antiviral drugs, immunotherapies, and vaccines with a diverse ranging limit of accuracy. Patients with early infection are mostly given antiviral agents to reduce viral load. However, patients with severe and late infections are given anti-inflammatory drugs [76]. These antibacterial and anti-inflammatory drugs are administered to prevent and treat probable secondary bacterial infections only. Hence, these cannot assure any direct or promised recovery from the virus itself. Besides, these drugs, potential vaccines, and therapies are still underdeveloped, raising the concern of creating various minor to severe side effects (Table 4). Moreover, the availability of these treatments is not yet reachable to many people depending on their geographical and economical differences. A selection of drugs and treatments being tested for COVID-19 is presented in Table 4.

**Table 4:** Possible non-specific treatments for the management of COVID-19

Non-specific treatments for the management of COVID-19					
Name	Target	Mode of Action	Mode of Administration	Potential Side Effects	References
Drugs					
Remdesivir	RNA-Dependent RNA Polymerase	Nucleotide analog	Intravenous	May damage liver	[77, 78]
				Nausea	
				Severe Headache	
Chloroquine/Hydroxychloroquine	ACE2	Heme polymerase inhibitor	Oral	Abdominal pain	[79–81]
				Diarrhea	
				Shortness of breath	
Lopinavir and Ritonavir	3CL protease	Protease inhibitor	Oral	Diarrhea	[82–84]
				Anemia	
				High blood pressure	
Favipiravir	RNA-dependent RNA polymerase	RNA polymerase inhibitor	Oral	Raised liver enzymes	[85–87]
				Hyperuricemia	
				QT prolongation	
Baricitinib	Janus kinase	JAK inhibitor	Oral	Sinus infections	[88, 89]
				Shingles	
				Urinary Tract Infection	
Nirmatrelvir	3C like protease	Covalent inhibitor of cysteine residue	Oral	Changes in taste, diarrhea, high blood pressure, or muscle pain	[167]
Molnupiravir	RNA-dependent RNA polymerase	RNA polymerase inhibitor	Oral	Diarrhea, dizziness, and nausea	[168]

Name	Developer	Type	Dose	Efficiency Rate	Potential Side Effects	References
*Vaccines*						
mRNA-1273	Moderna	mRNA	2	95%	Pain (91.6%)	[90, 91]
					Fatigue (68.5%)	
					Headache (63.0%)	
					Muscle pain (59.6%)	
					Joint pain (44.8%)	
BNT162b2	Pfizer & BioNTech	mRNA	2	95%	Fever	[90, 92]
					Chills	
					Tiredness	
					Headache	
AZD1222	University of Oxford & AstraZeneca	DNA (chimpanzee adenovirus vector)	2	62–90%	Myalgia	[90, 93]
					Nausea	
					Tenderness	
					Fever	
Sputnik V	Gamaleya Research Institute	DNA (human adenovirus vector)	2	92%	Flu-like symptoms	[90, 94]
					Pain at the injection site	
					Fatigue	

Name	Target	Mode of Action	Mode of Administration	Potential Side Effects	References
*Immunotherapies*					
Convalescent plasma therapy	Plasma from recovered SARS-CoV-2 patient	Producing SARS-CoV-2–specific IgG antibodies	Transplantation	May aggravate lung injury in patients with multiple organ failure	[95–97]
				May experience severe adverse reactions to blood transfusion	
Plasmapheresis	Blood component separator	Discarding pathogenic substances from patient’s plasma	Transplantation	Hypotension	[95, 98, 99]
				Risk of transfusion reactions	
				Suppression of the immune system	
Mesenchymal stem cell therapy	Inhibit excessive immune responses	Preventing uncontrolled mass production of cytokines or inflammatory factors	Transplantation	Tumorigenesis	[95, 100, 101]
				Genetic instability and chromosomal	

## Role of functional foods on respiratory and viral diseases

Respiratory viruses are the most frequent causes of human illness, which can cause respiratory tract infections that can range from mild to lethal [102]. To prevent respiratory tract infections, medicinal plants had been used for centuries in almost all cultures worldwide as traditional medicines. Leaves, roots, stems, fruits, flowers, and several other natural substances extracted from plants with therapeutic potential had been used to make herbal medicines. In addition, probiotics can have served as functional food supplements, which had the potential to cure several such health problems [103, 104].

Like other members of the coronavirus family, SARS-CoV-2 causes severe respiratory tract infections and chronic obstructive pulmonary diseases (COPDs). Previous studies have shown that dietary supplement solutions containing whey protein, carbohydrates, and antioxidants helped increase the human body's muscle strength by improving airflow limitation and reduced blood levels of inflammatory cytokines [105, 106]. In addition, it also showed that intake of vitamin E and D decreased the level of COPD risk, mortality rate, and increased inspiratory muscle strength [107, 108].

Several vitamins, including Vitamin C and E, have been reported to play a significant role in alleviating the immune response against respiratory viral agents [109, 110]. Respiratory infections deliberately lower the vitamin count from cells, negatively affecting the immune system causing severe diseases. Functional foods containing these vitamins have shown potential antioxidant effects against pulmonary diseases and prevented the severity of the infections [111, 112]. Moreover, fruits and vegetables containing β-Carotene and Lycopene have also shown free radical-scavenging activity and antioxidant effect against COPDs [113, 114]. Dietary supplements, vegetables, and fruits can also work over secondary bacterial infections due to coronavirus diseases, as they are important sources of nutrients, dietary fiber, and phytochemicals. A respiratory allergy experimental model has shown that probiotics induce a clear Th1 (elaborate) balance within the infected host favoring the production of IgG instead of IgE immunoglobulin and increasing the levels of IL-10 and IFN-γ cytokines, which reduces infection severity [115].

Medicinal plants were also used as supplements to treat SARS, MERS, and influenza viruses. Plant-based phenolic compounds from medicinal plants such as *Dioscorea batatas, Glycyrrhiza radix, Mollugo cerviana, Polygonum multiflorum Thunb., Psoralea corylifolia, Rheum officinale Baill., Salvia miltiorrhiza,* and *Trichosanthes cucumerina* L. were shown active against coronaviruses [116, 117]. Moreover, *Houttuynia cordata* has been found safe and effective for treating pneumonia caused by SARS-CoV and MERS-CoV [118]. A traditional Chinese medicine, Lianhuaqingwen, has been widely used to treat fever, cough, fatigue, influenza, bronchitis, pneumonia, early-stage measles [119, 120].

Another study revealed that *Q. Infectoria, B. integerrima, C. microphylla,* and *O. acanthium* extracts could exhibit ACE inhibition and antioxidant activities, which can be consumed as supplements [121]. These species could be promising sources of antiviral molecules that can decrease the reactive oxygen species (ROS) production in infected cells and target different oxidative stress-related signaling pathways resulting in a reduction in viral spread [122].

Therefore, functional food, dietary and herbal-based supplements have shown a huge impact in combating the symptoms of coronaviruses and increasing the potential activity of human immunity [123]. Furthermore, besides promising inhibitory effects, these food supplements are safe and efficient according to their consumption limit.

## Potential roles of functional foods for alleviating COVID-19

**Fig. 2:**
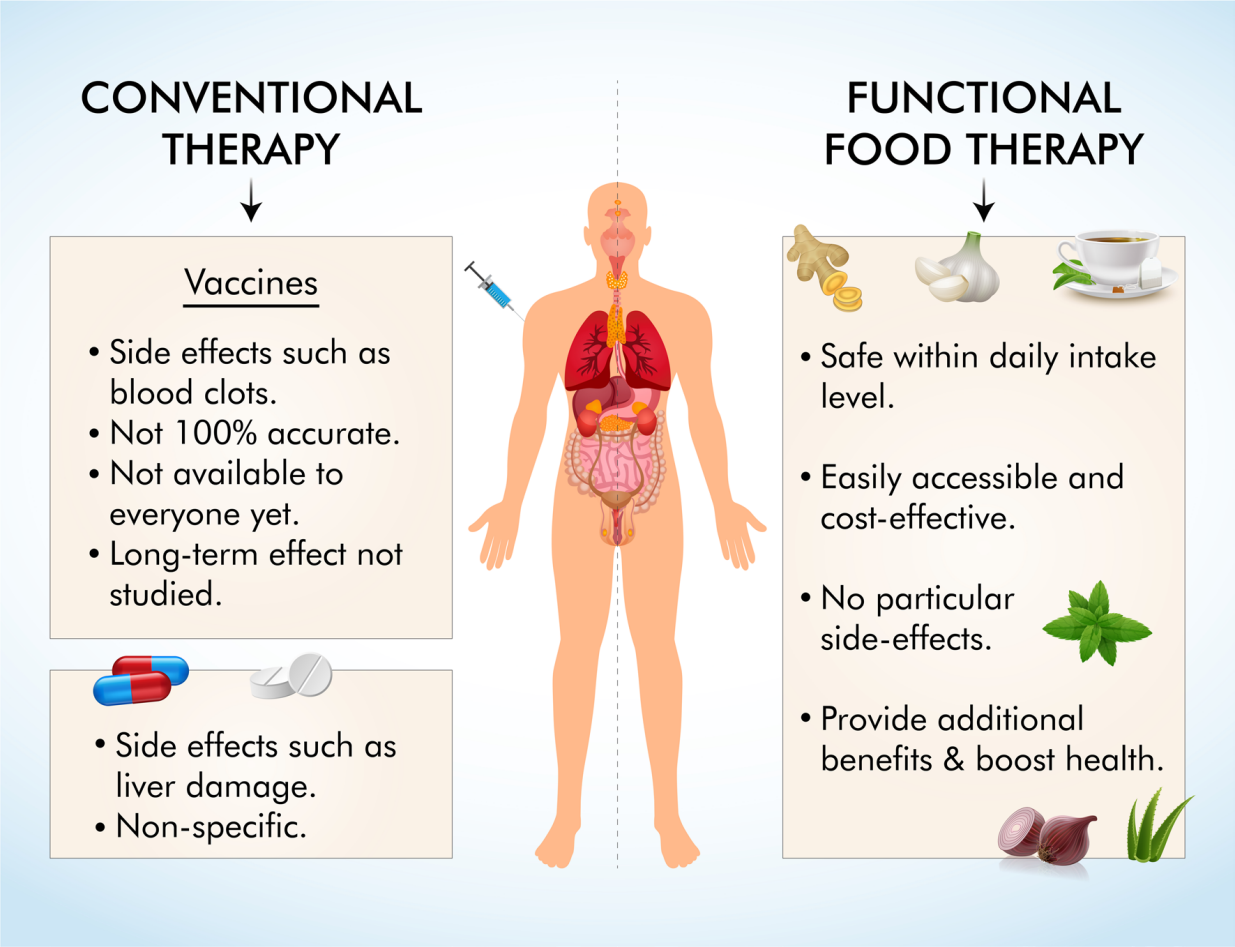
Comparison of conventional COVID-19 therapies with functional food therapy. Vaccines and drugs, both part of conventional therapy, pose many challenges such as side effects, inaccessibility, non-specificity, and inaccuracy. In addition, the long-term accuracy and side effects of all COVID-19 vaccines have not been studied, making vaccine treatment very uncertain. In contrast, functional foods are simple, cost-effective food ingredients that are available to everyone. Furthermore, since daily intakes for these foods have been studied rigorously and established, they are safe to consume while providing additional benefits and improving general health

A combination of few common foods is sufficient to prevent many diseases and even alleviate symptoms of patients. For example, herbs such as garlic could be easily added to daily meals, improving the utility of such dishes alongside palatability. For example, bioactive compounds in garlic can inhibit the main protease in SARS-CoV-2, reducing the spread of the virus inside the patient's body (Table 5). Also, the simple and easy addition of beverages such as green tea can be immensely helpful in the prevention of critical life-threatening diseases. Therefore, supplementing expensive drugs and therapy with cost-effective, simple food ingredients may be the best option for many patients, particularly those who cannot afford conventional medical treatments (Fig. 2).

**Table 5:** Different functional foods and their benefits outlined

Scientific name	Major Bioactive components examples	Properties	Role(s) in fighting COVID-19	References
*Allium sativum* (Garlic)	Allicin, ajoene, diallyl sulfide, diallyl disulfide, diallyl trisulfide, S-allyl-cysteine, S-allyl-cysteine sulfoxide (Alliin)	Anti-inflammatory	Alliin and other components inhibit the M^pro^ protease of SARS-CoV-2 by forming hydrogen bonds with it, thus inhibiting replication of the virus. Garlic increases the number of Treg cells, cytotoxic and helper T cells, and NK cells which are all reduced during COVID-19 infection. Garlic reduces leptin levels which improve appetite loss in COVID-19 patients.	[124–128]
		Antimicrobial		
		Anticancer		
		Anti-diabetic		
		Neuroprotective		
		Hepatoprotective		
		Anti-hypertensive		
		Cholesterol-lowering		
		Anti-obesity		
		Anti-hyperlipidemic		
*Zingiber officinale* (Ginger)	Gingerols (such as 6-gingerol), shogaols, paradols quercetin, zingerone, 6- dehydrogingerdione gingerenone-A, zingiberene, β-bisabolene, α-curcumene	Anti-inflammatory	8-Gingerol and 10-Gingerol inhibit SARS‑CoV‑2 main protease receptor by binding to its active site.	[125, 129, 130]
		Antimicrobial		
		Anti-diabetic		
		Neuroprotective		
		Anti-cholinergic		
		Anti-histaminic		
		Anti-obesity		
		Anti-nausea		
		Bioavailability enhancer		
*Curcuma longa* (Turmeric)	Curcuminoids (Curcumin)	Anti-inflammatory	Curcumin mediates anti-inflammatory responses against inflammatory cytokines such as IL-6, TNF-α, and IFN-γ. Thus curcumin may attenuate cytokine storms that prevent COVID-19 severity, such as ARDS. Reduced TNF-α also prevents pulmonary edema in COVID-19 lung disorders.	[125, 126, 131, 132]
		Antimicrobial		
		Anti-oxidative		
		Anticancer		
		Anti-diabetic		
		Immunomodulating		
		Anti-mutagenic		
		Radioprotective		
		Anti-tumor		
*Glycyrrhiza glabra* (Licorice)	Glycyrrhizin, glycyrrhizic acid, glabridin	Anti-inflammatory	Glycyrrhizin inhibits the 11bHSD2 enzyme, which degrades cortisol to increase aldosterone levels, leading to downregulation of ACE2 receptors and potential virus entry points in certain organs.	[125, 126, 133, 134]
		Anti-oxidative		
		Anticancer		
		Hepatoprotective		
		Immunostimulatory		
		Anti-pyretic		
		Anti-ulcer		
*Azadirachta Indica* (Neem)	Nimbolide, desacetylgedunin, azadirachtin, gedunin, azadirone, azadiradione, epoxyazadiradione, catechin, epicatechin	Anti-inflammatory	Desacetylgedunin, alongside 18 other compounds in Neem, has been shown in molecular docking studies to inhibit papain-like protease of SARS-CoV-2, which reduces viral spread.	[125, 135–137]
		Antimicrobial		
		Anti-oxidative		
		Anticancer		
		Anti-diabetic		
		Immunostimulant		
		Hepatoprotective		
		Neuroprotective		
		Anti-pyretic		
		Anti-ulcer		
		Anti-gingivitis		
*Ocimum sanctum* (Tulsi/Holy Basil)	Vicenin, Isorientin 4′-O-glucoside 2″-O-p-hydroxybenzoate, ursolic acid	Anti-inflammatory	Vicenin, Isorientin 4′-O-glucoside 2″-O-p-hydroxybenzoate and ursolic acid form bonds with M^pro^ protease of SARS-CoV-2 and inhibit it which would interfere with viral replication in patient.	[125, 126, 138, 139]
		Antimicrobial		
		Anti-oxidative		
		Anticancer		
		Immunomodulatory		
		Hepatoprotective		
		Anti-depressant		
		Anti-diarrheal		
		Anti-hypertensive		
		Analgesic		
		Adaptogenic		
*Piper nigrum* (Black pepper)	Piperdardiine, Piperanine, Piperlonguminine, Piperyline, Piperine, Piperettiine	Anti-inflammatory	Piperdardiine and Piperanine can inhibit SARS‑CoV‑2 main protease receptor by binding to its active site.	[130, 140]
		Antimicrobial		
		Anti-oxidative		
		Anticancer		
		Anti-depressant		
		Anticancer		
		Anti-parasitic		
*Cinnamomum zeylanicum* (Cinnamon)	Trans-cinnamaldehyde (TCA), cinnamaldehyde, eugenol, beta-caryophyllene, L-borneol, L-bornyl acetate	Anti-inflammatory	Cinnamon essential oil downregulates inflammatory biomarkers while cinnamon extract reduces the secretion of inflammatory cytokines such as IL-6 and TNF- α. This may result in the prevention of cytokine storm and COVID-19 complications such as lung fibrosis.	[126, 132, 141]
		Antimicrobial		
		Anti-oxidative		
		Anticancer		
		Anti-diabetic		
		Cholesterol-lowering		
		Lipid-lowering		
*Allium cepa* (Onion)	Quercetin, Ferulic acid, kaempferol, γ-glutamylcysteine, alliin, Zwiebelane A, Furfuraldehyde	Anti-inflammatory	Quercetin can improve COVID-19 associated pulmonary fibrosis through reduced collagen accumulation, inflammatory cell infiltration, alveolar wall thickness, and inflammatory markers.	[142, 143]
		Antimicrobial		
		Anti-oxidative		
		Anticancer		
		Anti-diabetic		
		Immunoprotective		
		Anti-hypertensive		
		Analgesic		
*Camellia sinensis* (Tea)	Epigallocatechin gallate (EGCG), epicatechin gallate (ECG), epigallocatechin (EGC), gallocatechin gallate (GCG),catechin (C), epicatechin (EC), gallocatechin (GC)	Anti-inflammatory	EGCG inhibits M^pro^ protease and structural proteins (6lu7, 6lvn, 6lxt, 6vsb, and 6vw1) better than conventional drugs in SARS-CoV-2.	[144, 145]
		Anti-oxidative		
		Anticancer		
		Neuroprotective		
		Anti-hypertensive		
		Lipid-lowering		
		Anti-photoaging

**Fig. 3:**
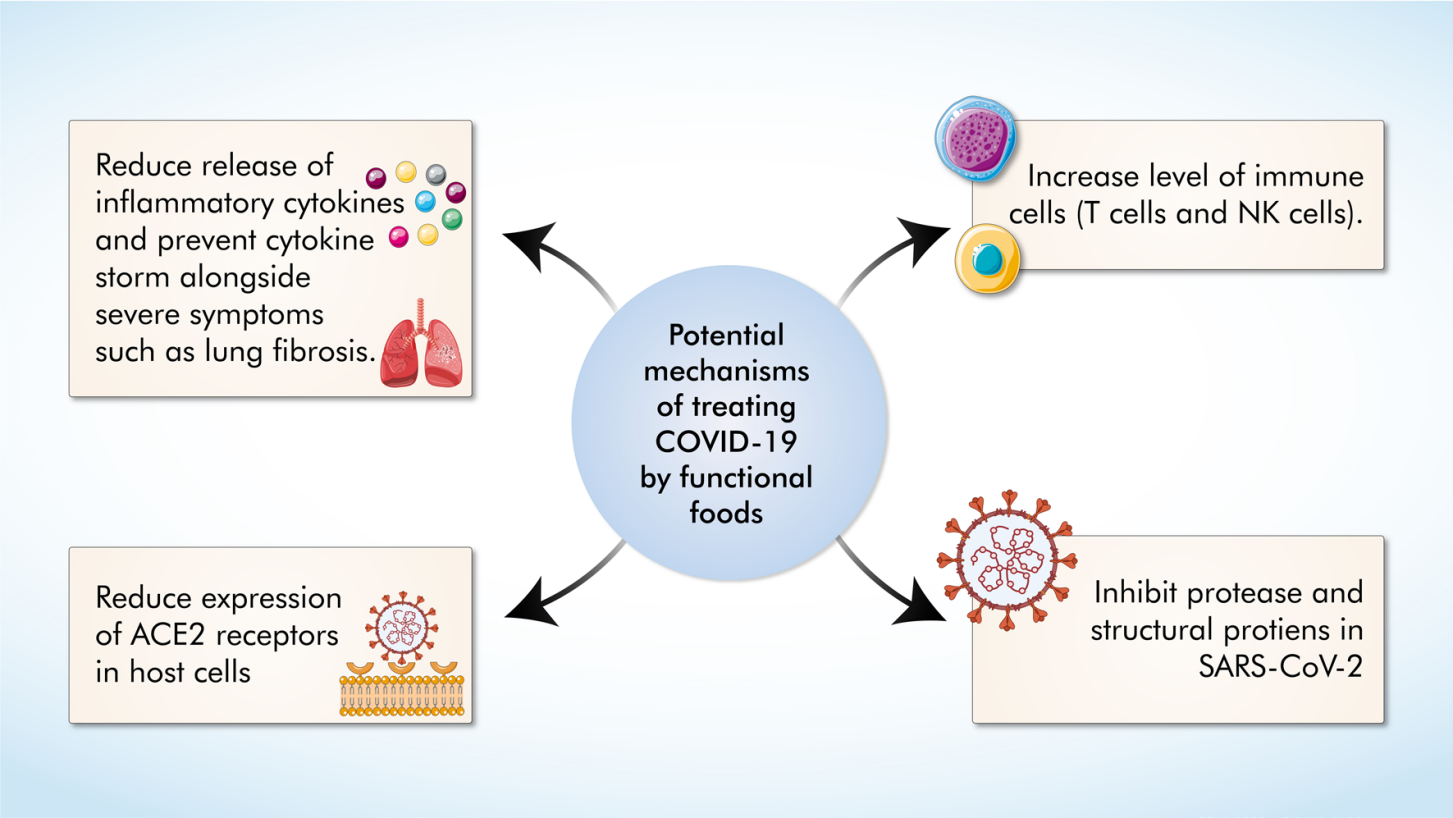
Proposed mechanisms of COVID-19 treatment by functional foods. Functional foods can alleviate COVID-19 symptoms through their immunomodulatory and antiviral properties. Preventing cytokine storms, elevating numbers of immune cells (T cells & NK cells), reducing ACE2 receptor expression, and inhibiting viral replication through inhibition of viral proteins and enzymes are some of the ways through which functional foods can help improve COVID-19 progression

Furthermore, intake of these simple foods is a good endeavor for avoiding many illnesses in the first place. While specific phytochemicals in these foods have the potential to mitigate COVID-19 (Fig. 3), large-scale clinical trials are required to propagate and justify the utilization of such foodstuffs for COVID-19 patients. A list of various functional foods is given in Table 5.

The severity of COVID-19 infections in patients consists of several key elements targeted with functional food as a therapeutic option instead of relying on costly medicines. One of the main symptoms of COVID-19 is a dry cough that is a nuisance to the patient and can propagate the virus particles into the air and other spaces, increasing the chance of infection. In some patients, coughing turns more severe and causes shortness of breath. Anti-inflammatory foods such as ginger can help reduce coughing. In addition, different compounds such as 6-gingerol and 8-gingerol relax the smooth muscles in the upper airways, reducing the incidence of coughing [146].

Additionally, Treg cells responsible for preventing cytokine storm alongside its associated complications are reported to be reduced in COVID-19 patients [147, 148]. COVID-19 patients have reported thrombotic complications associated with vitamin D deficiency. Therefore, consuming vitamin D-rich foods such as mushrooms, milk, and egg yolk can help prevent such complications. Minerals such as zinc also decrease COVID-19 infections that reduce autophagy and allow in vitro RdRp activity in SARS-CoV-2 [149]. Zinc-rich foods such as eggs modulate the function and abundance of immune cells alongside cytokine production, stimulate autophagy, improve antiviral drugs' efficacy, and inhibit the processing of viral polyproteins.

Secondary bacterial infections contracted from long-term clinical settings have been an important recurrence in hospitalized COVID-19 patients. Healthcare-associated infections coupled with less strict antibiotic stewardship approaches led to antibiotic-resistant pathogens in the ICU and other areas of the hospitals. Overall, Gram-negative bacteria were more prevalent in patients with the infectious agent varying according to the length of the hospital stay [150, 151]. In one hospital, *Klebsiella pneumoniae* was the predominant pathogen [151]. Garlic oil has demonstrated antimicrobial effects against *Klebsiella pneumoniae* and other pathogens [152]. Probiotics such as *Pediococcus pentosaceus* Li05 can be an effective prophylaxis to treat gastrointestinal infections [153].

Furthermore, SARS-CoV-2 alters redox homeostasis, accumulates excess reactive oxygen species (ROS), and produces oxidative stress in the body leading to lung and endothelial damage, cytokine storm, and insulin resistance [154]. Broccoli seeds were tested on COVID-19 patients, which quickly reduced cough, gastrointestinal symptoms, and other conditions associated with cytokine storm [155]. Nutrients interacting with nuclear factor (erythroid-derived 2)-like 2 (Nrf2), the most potent antioxidant, are present in various foods such as cabbage and fermented vegetables, which can downregulate the oxidative stress associated with COVID-19.

Importantly, post-COVID-19 complications can manifest in recovering individuals such as skeletomuscular symptoms (fatigue, muscle pain), gastrointestinal symptoms (diarrhea, nausea, vomiting), and neuronal symptoms (stroke, loss of taste/smell) [156]. Various functional foods can help alleviate these symptoms—*Astragalus radix* for fatigue; Sumac extract for muscle pain; zinc supplements for diarrhea, ginger for nausea and vomiting; omega-3 fatty acids for stroke, liposomal lactoferrin supplement for loss of taste/smell [157–161]. Finally, replication of SARS-CoV-2 can be prevented by inhibiting the essential protease enzymes, as demonstrated by molecular docking studies of compounds in garlic, ginger, Neem, tulsi, black pepper, and tea. In addition, foods such as licorice can downregulate ACE2 receptors, blocking viruses from entering cells (Table 5).

While many functional foods have been studied, countless foods targeting COVID-19 infections can be developed to form viable therapeutic options. For example, active ingredients in Rhizoma polygonati, an herb in traditional Chinese medicine, were screened from various databases to find 23 targets were found in SARS-CoV-2 by active ingredients [162]. Additionally, ten active compounds in Rhizoma Polygonati had good molecular docking scores with different drug targets of SARS-CoV-2 such as ACE2, 3CL hydrolase, Spike protein S1, and RNA-dependent RNA polymerase RdRp. Also, active compounds of Stingless bee honey demonstrate antibacterial activity (preventing secondary bacterial infection), antioxidant properties (reducing oxidative stress) and downregulation of IL-6 (shortening viral endurance in the body) [163]. Foods like this can be developed into novel functional foods through rigorous testing of efficacy, safety, and toxicity to produce viable, effective therapeutic options for COVID-19 infections.

## Conclusions

More than two years have progressed since the first case of the pandemic was contracted in Wuhan. Many countries are already experiencing multiple waves of COVID-19 with increasing strains on the health care system. As of June 2021, vaccines for COVID-19 have already been approved and distributed to millions of people worldwide, which has gradually reduced the infection rates in specific countries. However, the deployment of COVID-19 vaccines has raised concerns over equal, just access of different individuals to such an essential health care service. In December 2020, 51% of all vaccine doses were purchased by high-income countries that represent only 14% of the world population [164]. By April 2021, about 1 in 4 people were vaccinated in high-income countries compared to 1 in more than 500 in low-income countries [165].

Additionally, vaccines have been reported to have a low incidence of severe side effects in the population, such as Guillain–Barre syndrome (GBS), blood clots, and heart muscle inflammation [166]. Although such instances are rare, they have made the headlines that have instilled fear in many individuals who have become hesitant to receive the vaccine. Furthermore, antiviral drugs such as Remdesivir, which are used to treat COVID-19 infections, have harmful side effects that prolong the suffering of patients (Table 4). Therefore, until sufficient vaccines are supplied, alternative therapeutic options through functional foods must be employed. However, prescription of the diet with functional foods needs to consider toxicology studies as well. Toxicological evaluations reveal the necessity of further safety tests of different functional foods and standardizing their levels in a regular diet with the latest research data is essential. Furthermore, as the dosage of functional food has different effects on pre-existing conditions, specific safety levels for such individuals must be evaluated. Alongside functional foods, healthy lifestyle choices and regular exercises (that strengthen the immune system) can significantly reduce the financial strain of COVID-19, suffering of patients and death rates across the globe during this pandemic.

## Data Availability

Not applicable.
